# Antibody-Based Detection Tests for the Diagnosis of *Helicobacter pylori* Infection in Children: A Meta-Analysis

**DOI:** 10.1371/journal.pone.0003751

**Published:** 2008-11-18

**Authors:** Yelda A. Leal, Laura L. Flores, Laura B. García-Cortés, Roberto Cedillo-Rivera, Javier Torres

**Affiliations:** 1 Unidad de Investigación Médica Yucatán (UIMY), Unidad Médica de Alta Especialidad de Mérida, Instituto Mexicano del Seguro Social, Mérida, Yucatán, México; 2 Division of Pulmonary and Critical Care Medicine, San Francisco General Hospital and the University of California San Francisco, San Francisco, California, United States of America; 3 Unidad de Investigación Médica en Enfermedades Infecciosas y Parasitarias (UIMEIP)–Hospital de Pediatría Centro Médico Nacional Siglo XXI, Instituto Mexicano del Seguro Social, D.F., Mexico; Centre for DNA Fingerprinting and Diagnostics, India

## Abstract

**Background:**

Numerous serologic tests are available for the diagnosis of *H. pylori* infection in children. Common designs of antibody-based detection tests are ELISA and Western Blot (WB). For developing countries with limited laboratory resources and access, ELISA would be the preferred method because of its simplicity, lower cost and speed. Although in adults ELISA has proven to be highly accurate in diagnosing *H. pylori* infection; in children, it has shown variable accuracy.

**Methods/Findings:**

We conducted a systematic review and meta-analysis to assess the accuracy of antibody-based detection tests for the diagnosis of *H. pylori* infection in children. Selection criteria included participation of at least 30 children and the use of a gold standard for *H. pylori* diagnosis. In a comprehensive search we identified 68 studies. Subgroup analyses were carried out by technique, immunoglobulin class, and source of test (commercial and in-house). The results demonstrated: 1) WB tests showed high overall performance, sensitivity 91.3% (95% CI, 88.9–93.3), specificity 89% (95% CI, 85.7–91.9), LR+ 8.2 (95% CI, 5.1–13.3), LR− 0.06 (95% CI, 0.02–0.16), DOR 158.8 (95% CI, 57.8–435.8); 2) ELISA-IgG assays showed low sensitivity 79.2% (95% CI, 77.3–81.0) and high specificity (92.4%, 95% CI, 91.6–93.3); 3) ELISA commercial tests varied widely in performance (test for heterogeneity p<0.0001); and 4) In-house ELISA with whole-cell antigen tests showed the highest overall performance: sensitivity 94% (95% CI, 90.2–96.7), specificity 96.4% (95% CI, 94.2–97.9), LR+ 19.9 (95% CI, 7.9–49.8), LR− 0.08 (95% CI, 0.04–0.15) DOR 292.8 (95% CI, 101.8–841.7).

**Conclusions/Significance:**

WB test and in-house ELISA with whole-cell antigen tests are the most reliable tests for the diagnosis of *H. pylori* infection in children. Antigens obtained from local strains of the community could partially explain the good overall accuracy of the in-house ELISA. Because of its cost and technical demands, in-house ELISA might be more suitable for use in developing countries.

## Introducion

*Helicobacter pylori* infection is one of the most common bacterial infections in humans affecting nearly 50% of the world's population [1], [2]. *H. pylori* has been linked with the development of gastritis, peptic ulcer, gastric cancer and MALT-lymphoma [3]–[5]. Infection is usually acquired during childhood and is associated with socio-demographic factors such as low socio-economic status, poor hygiene, and crowding [6]–[8]. In children the diagnosis is often difficult to establish as signs and symptoms; such as abdominal pain, diarrhea, and occasional vomiting, are non-specific [9]–[11]. *H. pylori* colonisation results in local and systemic humoral response and it has been reported that, after infection, children primarily develop an immune serologic response against low-molecular-weight antigens [12]–[14].

Currently, several invasive and non-invasive diagnostic tests are used for the detection of *H. pylori* infection in children [10], [11], [15], [16]. Invasive tests rely primarily on the identification of *H. pylori* in culture, histological examination, and the rapid urease test (RUT) [11], [17], [18]. Non-invasive tests include the detection of bacterial urease activity by urea breath test (UBT), antibody-based detection test in different fluids; and recently antigen detection in stool [11], [19]–[23].

Serological tests for *H. pylori* infection have been helpful in epidemiological studies of prevalence, mode of transmission and spontaneous clearance of the infection; allowing for the development of preventive measures of infection early in life [6], [7], [24], [25]. Antibody-based tests have been developed during the last decades. These tests differ in a number of features: antigen composition (e.g., different *H. pylori* strains); antigen source (e.g., native or recombinant); protocols for antigen purification; class of immunoglobulin detected (e.g., IgG, IgA, IgM); origin of samples (e.g., serum, saliva, urine); and test source (i.e., commercial and in-house test). The main advantages of antibody-based tests are their simplicity, low cost, speed, and minimal patient discomfort. Their performance has been critically appraised in several descriptive reviews and textbook chapters [26]–[30]. Common designs of antibody-based detection test include the enzyme-linked immunosorbent assay (ELISA) and the Western Blot (WB) technique. The ELISA format has the advantages that many serum samples can be tested in parallel and the process can be completely automated. For developing countries with limited laboratory resources and access, this test would be the preferred method. Although ELISA has proven to be highly accurate for the diagnosis of *H. pylori* infection in adults, in children it has shown variable sensitivity and specificity [10], [11], [15], [16], [31], [32]. On the other hand, WB allows the direct visualization of antibody binding to specific *H. pylori* antigens, including the virulence factors CagA and VacA [33]. These proteins are highly immunogenic and usually stimulate a specific IgG immune response against proteins of 118 to 136 kDa for CagA and 89 kDa for VacA. Hence, this test seems to be more accurate for the diagnosis of *H. pylori* infection in children, and presumably could additionally distinguish infection with virulent strains [14], [34], [35].

The systemic immune response against *H. pylori* typically shows a transient rise in specific IgM antibodies, followed by a rise in IgG and IgA antibodies that persist during infection. Since IgM antibodies against *H. pylori* are detected only transiently, they have little value for the serological diagnosis of infection [27]. Therefore diagnostic commercial and in-house tests have been developed for detection of *H. pylori*-specific IgG and IgA antibodies in serum saliva and urine. Detection of *H. pylori*-specific IgA and IgG antibodies in saliva from general population has shown limited sensitivity 80% and specificity 70% [28], [36]–[37]. *H. pylori*-specific IgG in urine has shown more sensitivity (95.5%) and specificity (83%) [38]–[40]. Most IgG diagnostic tests are serum-based. Several studies have evaluated the diagnostic value of commercial ELISA-IgG for the detection of *H. pylori* infection. Two previous meta-analyses performed in general population have been described. The first meta-analysis (21 studies) only evaluated commercial ELISA tests and reported an overall sensitivity and specificity of 85% and 79%, respectively [41]. The second meta-analysis (36 studies) assessed the performance of different commercial *H. pylori* tests measuring IgA, IgG and IgM antibodies and reported pooled estimates for sensitivity of 92% and for specificity of 83%. Overall accuracy was low and considerable heterogeneity was present, these values of sensitivity and specificity reported in the previous meta-analysis reflect the response values mostly in adults [42].

When the performance of a diagnostic test is evaluated, properties of the test are often described using sensitivity and specificity. The addition of statistics such as positive and negative likelihood ratios (LR+ and LR−) and the diagnostic odds ratio (DOR) can help the healthcare provider determine how to interpret the result of the test in a more clinically meaningful way for the pediatric patient [43]–[45]. Hence, we carried out a systematic review and meta-analysis to evaluate the performance of the different antibody-based detection tests available for *H. pylori* infection in children by determining sensitivity and specificity estimates as well as additional accuracy values relevant to clinical practice.

## Methods

### Identification of studies

We searched the databases PUBMED, EMBASE, and LILACS for references published between January 1997 and May 2007. The search terms used included: “*Helicobacter pylori*” “Children”, “Serological Test”, “Antibody Detection”, “Western Blot”, “ELISA”, “Specificity” and “Sensitivity”. English and Spanish references were included in the search. The final set of in-extent review of the selected literature included cross checked references and direct communication with the corresponding authors when the article was not available in full length on-line.

### Study eligibility and data extraction

Studies were initially selected according to the following criteria: a) language: English or Spanish full text articles; b) diagnosis of *H. pylori*: based on gold-standard (culture, histology and/or UBT); c) study design: cross-sectional or case control; d) data collection: prospective or retrospective; e) sample size: at least 30 participants (15 patients and 15 controls); f) age: 0–19 year; and g) data: actual numbers of True Positive, True Negative, False Positive and False Negative results of the tests or predicted positive and negative values. The articles that were finally included in the meta-analysis were reviewed independently by two different experts and discrepancies in the interpretation were resolved by consensus. Data was included in an Excel database which was cross checked for input errors. With the information available in the selected studies, we calculated the following values: PPV, PPN, LR+, LR−, DOR and their corresponding 95% confidence intervals (95%, CI). Reviews, letters to the editor, opinions and recommendations about the diagnostic *H. pylori* infection in children were excluded.

### Assessment of Study Quality

We assessed the quality of the studies using the following criteria, which have been suggested as being important for diagnostic studies [46]; a) was there a comparison of the antibody-based detection test with an appropriate reference standard? (i.e. the ELISA and WB detection tests did not form part of the reference standard); b) was the antibody-based detection test result performed and recorded by technicians who were unaware (i.e. blinded) of the results of the reference standard?; c) did the whole sample or a randomly selected subset of the sample received verification using the reference standard?; d) did the study prospectively recruit consecutive children suspected of having *H. pylori* infection? (i.e. cross-sectional versus case control design).

### Outcomes of interest

We determined sensitivity (proportion of positive test result among those with the infection) and specificity (proportion of negative test results among those without the infection). In addition, we calculated LR's statistics considered to provide guidance to clinicians. LR+ measures how many times a positive test is more likely found in infected versus non-infected children, whereas LR− measures how many times a negative result is more likely found in infected versus non-infected children. A higher value of the LR+ confirms the presence of the infection; and a lower LR− excludes the presence of the infection, in contrast to the positive and negative predicted values (PPV & NPV), the LR's allow the determination of the accuracy of the test in populations with different prevalence of the infection. Furthermore, LR+ and LR− are combined to obtain a new factor, DOR that describes the ratio of the odds of a positive result test in a child with infection compared with a positive test in a child without infection. The value of a DOR ranges from 0 to infinity, with higher values indicating better discriminatory performance or higher accuracy of the test [43]–[45].

### Meta-analysis

We used standard methods recommended for meta-analysis of diagnostic tests studies [47], [48]. Estimates of sensitivity and specificity from individual studies and their exact 95% confidence intervals were obtained and forest plots made using MetaDiSc Beta-1.4 software (Universidad Complutense, Madrid España).

#### Heterogeneity

In meta-analysis of diagnostic studies heterogeneity refers to the degree of variability in accuracy estimates across studies. An exploration of the reasons for heterogeneity is an important goal of a meta-analysis [49]. When significant heterogeneity is present, summary measures of test accuracy are difficult to interpret. Statistical significance of heterogeneity among studies was calculated using the chi-square test. We further investigated reasons for heterogeneity using stratified (subgroup) analyses.

#### Summary Receiver Operating Characteristic (SROC) Curve

We summarized the joint distribution of true positive and true negative rates in a SROC curve. The SROC curve is used for the evaluation of diagnostic tests and represents the relationship between true positive and negative rates considering the varying diagnostic thresholds among studies. Each data point in the SROC space indicates the sensitivity and specificity estimates of a single study. A regression curve is fitted through the distribution of the paired sensitivity and specificity values. The area under the curve (AUC) represents an analytical summary of test performance and display the trade-off between sensitivity and specificity. An AUC of 1.0 (100%) indicates perfect discriminatory ability to distinguish cases from non cases. The Q* index, is the highest point in the SROC curve that intersects the anti-diagonal and represents a summary of test performance where sensitivity and specificity are equal. A Q* index of 1.0 indicates 100% or perfect accuracy (sensitivity and specificity of 100%). Both values range between 0 to 1, and higher values indicate better test performance [43], [50], [51].

## Results

### Study selection

**Figure 1:**
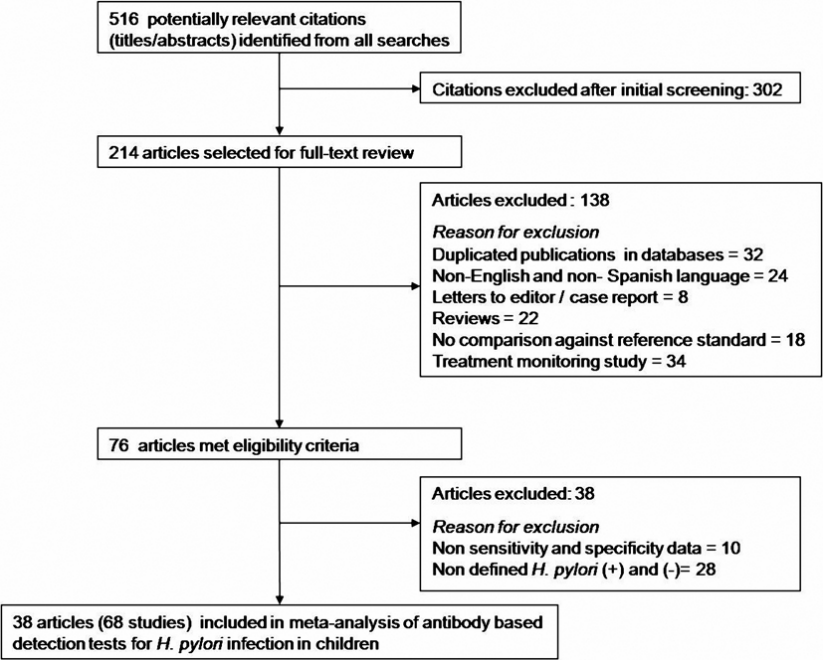
Study selection process and reasons for exclusion.

The search of the selected databases retrieved 516 potentially relevant references on diagnostic tests for *H. pylori* infection in children. After screening titles and abstracts, 214 English and Spanish articles were selected for full-text review, and 76 of them met the eligibility criteria; from these articles ten were excluded because data did not provide reliable information about sensitivity and specificity; and other 28 articles were excluded due to lack of a gold standard for diagnosing *H. pylori* infection. In the end, 38 articles (68 studies) met eligibility criteria and were included in the meta-analysis [52]–[89]. Figure 1 shows the study selection process.

### Characteristics of included studies

Of the 38 articles, 18 [52], [53], [56], [58], [59], [61], [62], [67], [69], [71], [73], [74], [76], [78], [79], [82], [85], [89] (47.5%) used culture and histological examination as the gold standard; ten [54], [60], [64], [70], [72], [80], [81], [83], [84], [87] (26.3%) used histology in combination with RUT and UBT; two articles [63], [68] (5.2%) used histology alone and seven articles [55], [57], [66], [75], [77], [86], [88] (18.4%) used UBT; and finally one article [65] (2.6%) used culture + UBT as gold-standard. For all articles the ELISA and WB tests did not form part of the reference standard. The median sample size of the included studies was 110.5 (interquartile range 75.8 to 184). Three papers in Spanish were included [62], [64], [76]. Thirty-one (81.5%) articles reported using a case-control design and the remaining seven [53], [57], [65], [66], [68], [69], [75] (18.5%) a cross-sectional design. Thirty-two of the 38 (84.2%) articles collected samples prospectively and six [53], [65], [66], [69], [74], [85] (15.8%) retrospectively; two [65], [75] (5.2%) articles reported the use of a randomly selected subset of the sample for validation with the reference standard. Eleven [52], [61], [63], [64], [67], [68], [78]–[81], [83] (28.9%) articles reported at least single-blinded interpretation of the ELISA and WB tests and reference standard results, while 27 (71.1%) articles did not mention the blinding status. Thirteen (34.2%) of the 38 articles reported evaluation of more than one diagnostic test against the gold-standard; in these cases each test comparison was counted as a separate study. Thus, the total number of test comparisons (hereafter referred to as studies) was 68. A total of 9,455 children were included in the meta-analysis. Of these; 3,441 were *H. pylori* positive and 6,014 were *H. pylori* negative according to the gold standard. Clinical specimens evaluated included serum, urine, and saliva. For the meta-analysis the serological antibody detection tests were divided into two groups: ELISA and Western Blot. ELISA group was further sub-grouped by type of sample: serum, urine, or saliva. The serum subgroup was divided into IgG and IgA according to the immunoglobulin isotype that was determined. Table 1 describes the characteristics of the 68 studies and the outcomes for the subgroups of diagnostic tests.

**Table 1:** Summary of characteristics of the studies included in the meta-analysis.

Diagnostic Test	No Studies	Sample Size (*H. pylori*+/−)	Test Type	DOR (95% CI)	*Test for Heterogeneity
Serum					
ELISA-IgG	42	1861/3771	+CK (33)	60.9	127.2 (<0.0001)
			In-house (9)	(41.8–88.6)	
ELISA-IgA	7	250/329	CK (3)	9.6	9.4 (0.150)
			In-house (4)	(4.8–19.0)	
Western-Blot	10	699/420	CK (8)	158.8	27.6 (0.001)
			In-house (2)	(57.8–435.8)	
Urine					
ELISA-IgG	4	301/437	CK (4)	44.2	5.9 (0.116)
				(17.2–113.9)	
Saliva					
ELISA-IgG	5	330/1057	CK (5)	49.1	12.8 (0.012)
				(22.6–106.8)	
**Total**	**68**	**3441/6014**	**------**	**-------**	**------**

+ CK = Commercial Kit.

* Chi-squared and *p* value.

### How accurate is ELISA for the diagnosis of *H. pylori* infection in children?

Fifty-eight studies, involving a total of 8,336 children (2,742 *H. pylori* positive and 5,594 *H. pylori* negative), assessed the diagnostic accuracy of the ELISA test. Table 1 shows performance and other characteristics for this group.

#### Serum

**Figure 2:**
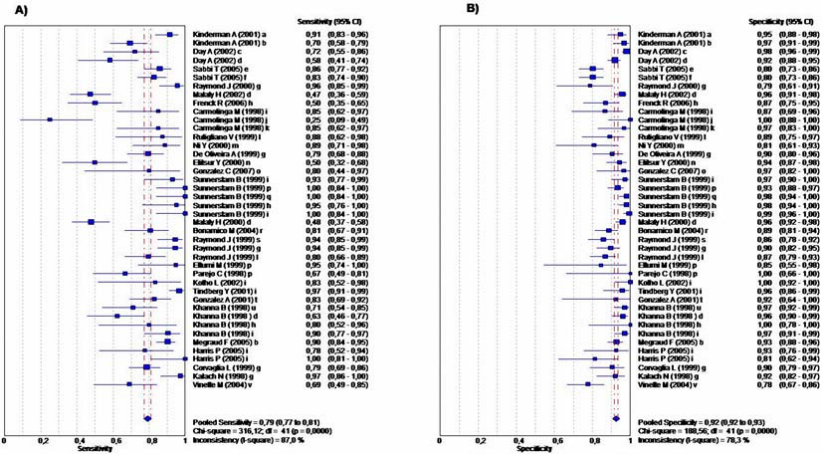
Forest Plot for the Sensitivity and Specificity of the Serum IgG-ELISA Diagnostic Tests. The squares and lines represent the point estimates and 95% CIs, respectively. The size of square indicates the study size. The pooled estimated is denoted by a diamond at the bottom. *a = Enzygnost II, b = Pyloritest, c = MedMira; d = FlexSure, e = Eurospital, f = Eurospital-CagA, g = Cobas II, h = HM-CAP, i = In-house WC, j = In-house Urease, k = In-house CagA, l = GAP-Test, m = HEL-p II, n = Cobas I, o = InmunoLISA, p = Helico-G, q = Pyloriset, r = Helory, s = Platelia, t = Immulite, u = PyloriStat, v = GAP-Biomerica*

**Figure 3:**
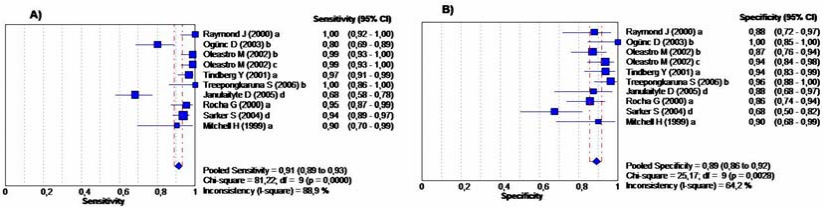
Summary Receiver Operator Curve (SROC) for Serum IgG-ELISA Diagnostic Tests. Each solid square represents an individual study in the meta-analysis. The curve is the regression line that summarizes the overall diagnostic accuracy. AUC = area under curve, SE (AUC) = standard error of AUC, Q* = index defined by point of the SROC curve where the sensitivity and specificity are equal; SE (Q*) = standard error of Q* index.

Forty two studies were included in the subgroup for ELISA-IgG, of these, 33 (78.5%) studies assessed the performance of 19 different commercial tests, and nine (21.4%) studies assessed the performance of in-house test. These tests used different *H. pylori* antigens, such as Whole Cell (WC), Urease, VacA, and CagA recombinant protein. Regarding commercial tests, Cobas Core EIA® (Roche, Mannheim, Germany) and FlexSure®HP Serum Test (SmithKlineDiagnostics Palo Alto, CA, USA) were the tests most frequently evaluated (Figure 2A y B). Four of the seven studies included in the ELISA-IgA subgroup used in-house tests (two of them used CagA or Urease recombinant protein as antigen), and the other three used commercial tests. The ELISA-IgG test subgroup included 4,781 children; a summary of the accuracy measures of this test is shown in Table 2. Pooled estimates of sensitivity, specificity, LR+, LR−; were 79.2%, 92.4%, 10.2, and 0.19 respectively. The results of the serum ELISA-IgG subgroup (42 studies) are shown in Figure 2A. In general, specificity estimates were higher and more consistent (range 78% to 100%) than sensitivity estimates (range 25% to 100%). The corresponding SROC (Figure 3) shows an area under curve of 0.95 and a Q* of 0.89, indicating high overall accuracy. The pooled DOR was 60.9 (95% CI, 41.8–88.6), but heterogeneity across studies was significantly high (p<0.0001) (Table 1). When compared with the ELISA-IgG subgroup, the ELISA-IgA subgroup provided lower estimates for sensitivity, specificity, LR+ and LR− 42.6%, 90.9%, 4.4, and 0.60 respectively. In addition, the accuracy (AUC = 0.85 and Q* = 0.78) and DOR value 9.6 (95% CI 4.8–19.0) were lower for ELISA-IgA than for ELISA-IgG, although in this case heterogeneity was not significant (p = 0.150) (Table 1).

**Table 2:** Summary measures of test accuracy from the studies included.

Diagnostic Test	Sensitivity	Specificity	LR+	LR−
	(95% CI)	(95% CI)	(95% CI)	(95% CI)
Serum				
ELISA-IgG	79.2 (77.3–81.0)	92.4 (91.6–93.3)	10.2 (8.1–13.0)	0.19 (0.15–0.25)
ELISA-IgA	42.6 (36.4–49.0)	90.9 (87.2–93.8)	4.4 (2.7–7.1)	0.60 (0.45–0.79)
Western-Blot	91.3 (88.9–93.3)	89.0 (85.7–91.9)	8.2 (5.1–13.3)	0.06 (0.02–0.16)
Urine				
ELISA-IgG	59.1 (53.3–64.7)	92.9 (90.1–95.1)	9.6 (3.9–23.4)	0.23 (0.08–0.68)
Saliva				
ELISA-IgG	69.1 (63.8–74.1)	94.7 (93.2–96.0)	14.4 (7.3–28.6)	0.33 (0.28–0.39)

LR+ = Likelihood positive.

LR− = Likelihood negative.

CI = Confidence Intervals.

#### Urine and Saliva

We assessed four studies in the urine group and five in the saliva group, including 738 and 1,387 children respectively. Table 1 shows the performance and characteristics of these studies; all of them evaluated commercial tests and determined the IgG isotype. The antigen composition was not described because it was considered proprietary information. Summary measures of test accuracy for both sample sources were: for the urine group sensitivity, specificity, LR+ and LR− were 59.1%, 92.9%, 9.6 and 0.23 respectively; whereas for the saliva group values were 69.1%, 94.7%, 14.4 and 0.33 respectively. The sensitivity estimates were lower and more variable than the specificity estimates in both sample groups (Table 2). Furthermore this variation was more notable in the urine group (range 30% to 94%) than in the saliva group (range 65% to 81%). However, the overall accuracy of the tests was higher in the urine group AUC = 0.94, and Q* = 0.88 than in the saliva with AUC = 0.85, and Q* = 0.78 indicating a modest accuracy for the latter (Data not shown). The pooled DOR value was similar for the two groups (urine: 44.2 [95% CI, 17.2–113.9]; saliva 49.1 [95% CI, 22.6–106.8]). Only for the saliva group, was significant heterogeneity present (p = 0.012) (Table 1).

### How accurate is Western Blot for the diagnosis of *H. pylori* infection in children?

**Figure 4:**
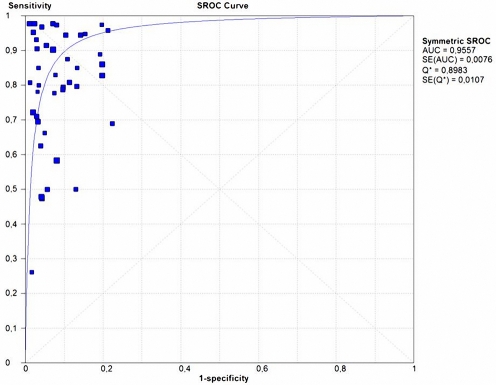
Forest Plot for the Sensitivity and Specificity of the Western Blot Diagnostic Tests. The squares and lines represent the point estimates and 95%CIs, respectively. The size of square indicates the study size. The pooled estimated is denoted by a diamond at the bottom. *a = HelicoBlot 2.0, b = HelicoBlot 2.1, c = HelicoBlot 2.1 CIM; d = In-house*

**Figure 5:**
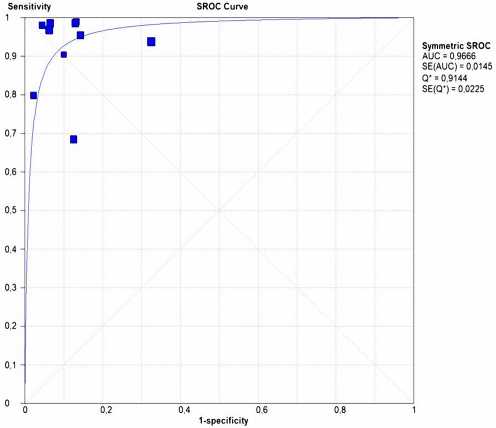
Summary Receiver Operator Curve (SROC) for Western Blot Diagnostic Tests. Each solid square represents an individual study in the meta-analysis. The curve is the regression line that summarizes the overall diagnostic accuracy. AUC = area under curve, SE (AUC) = standard error of AUC, Q* = index defined by point of the SROC curve where the sensitivity and specificity are equal; SE (Q*) = standard error of Q* index.

Ten studies were included in the Western Blot group involving 1,119 children between 1 and 16 years of age. Eight of the ten studies used HelicoBlot® (Genelabs Diagnostics, Singapore), a commercial test that is based on the analysis of whole-cell *H. pylori* antigens. Currently, there are two presentations available: version 2.0 contains antigens of 19.5, 26.5, 30, 35, 89 (VacA), and 116 (CagA) kDa; and version 2.1 contains antigens of 19.5, 30, 35, 37, 89 (VacA), and 116 (CagA) kDa, the latter version also contains an additional recombinant antigen (∼45 kDa) named the *current infection marker* (CIM). This protein was constructed by immunological screening of a genomic DNA library of *H. pylori* (ATCC strain 43526) [90]. Four studies used HelicoBlot version 2.0, four used version 2.1, and two evaluated the performance of in-house Western blot test (Figure 4A and B). Summarized accuracy measures are shown in Table 2. Pooled estimates of sensitivity, specificity, LR+, LR−; were 91.3%, 89.0%, 8.2, and 0.06 respectively. The sensitivity and specificity estimates were similar in range and varied between 69 to 100% for sensitivity and between 68 to 100% for specificity (Figure 4A). Figure 5, shows the corresponding SROC curve; the area under the curve was 0.96 and the Q* was 0.91, indicating a high overall accuracy. In addition, the studies that evaluated Western Blot test had a high DOR value 158.8 (95% CI, 57.8–435.8), nevertheless heterogeneity was significant (p = 0.001) (Table 1).

### Possible reasons for the observed heterogeneity

**Figure 6:**
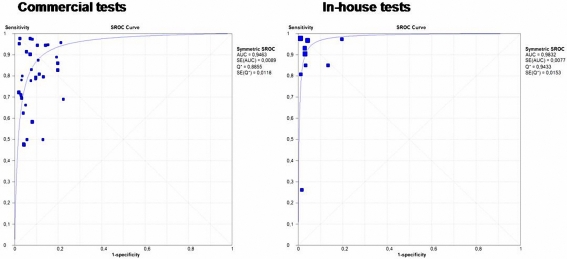
Summary Receiver Operator Curves (SROC) for commercial and in-house tests from Serum IgG-ELISA. Each solid square represents an individual study in the meta-analysis. The curve is the regression line that summarizes the overall diagnostic accuracy. AUC = area under curve, SE (AUC) = standard error of AUC, Q* = index defined by point of the SROC curve where the sensitivity and specificity are equal; SE (Q*) = standard error of Q* index.

In order to identify factors associated with the considerable heterogeneity observed in the serum ELISA-IgG group, we stratified this group into two subgroups, commercial (33 studies) and in-house (9 studies) tests and compared their performance. Since there was a large number (19 tests) of different commercial tests evaluated in this review, greater heterogeneity was expected. In-house tests showed high overall performance with pooled estimates of sensitivity 88.4%, specificity 96.6%, LR+ 19.7, and LR− 0.10. Also, when we performed the analysis only with whole-cell antigen for the in-house test the overall performance was better with pooled estimates of sensitivity 94%, specificity 96.4%, LR+ 19.9, and LR− 0.08 as compared with the estimates for the commercial tests 77.7%, 91.8%, 9.2, and 0.22 respectively. Again, the overall accuracy of the in-house tests with whole-cell antigen was higher (AUC = 0.98 and Q* = 0.94.2) than that of the commercial tests (AUC = 0.94 and Q* = 0.88) (Figure 6). Furthermore, heterogeneity prevailed only in the commercial test group, DOR value was 46.9 (95% CI, 32.4–67.9) and heterogeneity was significant (p<0.0001). In contrast, in-house test had higher DOR value 224.8 (95% CI; 87.5–577.5); and in-house ELISA with using exclusively whole-cell antigen had the highest DOR value 292.8 (95% CI; 101.8–841.7); in both cases a non-significant heterogeneity was documented (p = 0.119).

## Discussion

### Principal findings

Our comprehensive literature search identified 68 studies that addressed performance of tests for the diagnosis of *H. pylori* infection in children. The results of the meta-analysis suggest that (1) WB tests show high overall performance with sensitivity of 91.3%, specificity of 89%, reasonably high LR+ (8.2) and very low LR− (0.06), suggesting WB is the most reliable test for the diagnosis of *H. pylori* infection in children; (2) ELISA-IgG assays provide low sensitivity (79.2%) and high specificity (92.4%); (3) ELISA commercial tests vary widely in performance (test for heterogeneity p<0.0001); and (4) In-house ELISA with whole-cell antigen tests have high accuracy (sensitivity 94% and specificity 96.4%). This is also reflected in the high LR+ estimate (19.9) and low LR− estimate (0.08), suggesting that these tests have a potential role in ruling out or confirming *H. pylori* infection in children.

Potential explanations for the lower specificity of WB-based tests include the following: a) the occurrence of transient *H. pylori* infection (spontaneous clearance of the infection) which has been reported as a common phenomenon in children. This observation may explain the incidence of false positive results since antibody titers decrease slowly after clearance of infection [24], [91], [92]; b) immunological cross-reaction, as the flagellar complex protein has shown antigenic similarity with spiral-shaped bacteria colonizing the intestinal mucus, such as *C. jejuni* [93], [94], and c) variable criteria for results interpretation, stemming from different published recommendations which have resulted in considerable confusion regarding interpretation criteria [95]–[98]. For example, one interpretation system used a quantitative cutoff value to assess band intensity [98], while other systems considered any band intensity as a positive result [96], [97]. Several interpretation systems do not use criteria for low molecular weight and weak bands, which represent the two components of the flagellar complex (55 and 59 kDa) [95], [98]. An LR+ of 8.2 for WB-based tests provides moderate evidence that children with *H. pylori* infection have a greater chance of being WB test positive, compared with children without the infection. This ratio is a reasonable guide enabling the clinician to rule in (e.g. confirm) infection [99]. Also, an LR− of 0.06 provides strong evidence to exclude *H. pylori* infection when a child's WB test result is negative. This result is important since it guides the clinician to refrain from unnecessary and potentially harmful treatments of children who are not actually infected.

It is generally accepted that *H. pylori* infection is acquired in childhood or adolescence, and that early acquisition of this infection could increase the risk of *H. pylori*-related complications later in life; in fact it has been proposed that earlier infection is linked to a greater risk for gastric cancer [3], [4], [8]. These risks for disease during adulthood are good reason to have an accurate diagnosis of *H. pylori* infection in childhood. Although a number of authors have studied the antibody response against *H. pylori* in children, the results reported have been variable, which may be explained partially by the variation in prevalence across populations and age-groups [1], [2], [6]–[8]. Still, in many instances the variability of results may also be due to differences in the characteristics of the diagnostic tests used.

Regarding ELISA for the diagnosis of *H. pylori* in serum, urine and saliva from children, our results suggest that detection of IgG antibodies in serum is acceptable, with high specificity 92.4% and LR+ of 10.2 indicating that children with *H. pylori* infection have 10.2 fold higher chance of being ELISA-IgG test positive compared with non-infected children. The summary estimate for sensitivity, however, was lower 79.2%, and more variable than the specificity estimate. Furthermore, antibody detection in urine and saliva samples yielded the lowest sensitivities, 59.1% and 69.1%, respectively, suggesting that these tests did not perform well in children. The corresponding LR− ratios, (urine, 0.23 and saliva 0.33) suggest that infection cannot be excluded when test results are negative. The amount of antibodies in urine and saliva probably correlates with the amount of antibodies present in serum, but at a lower concentration, possibly explaining why these samples provided lower sensitivities than serology. The low sensitivity in antibody detection by ELISA may also be explained by: a) age. Our meta-analysis included children with a wide range of ages (<1 to 19 years). The ability to mount an efficient immune response varies with age, showing a weaker response during the first years of life [10], [13], [14], [78]; b) ethnic groups. Different *H. pylori* strains and even different antigens of the same strain show diverse antigenicity among ethnic groups and geographical areas. Sequence heterogeneity in protein-encoding genes may result in variation of immunogenic epitopes [29], [33], [100]; c) specimen handling. The majority of studies used frozen sera; thus samples from different studies were subjected to diverse freeze-thaw cycle histories which may affect sensitivity [27], [30]; d) reference standard. Although culture and histology are considered gold standards for diagnosing *H. pylori* infection, these tests are not 100% accurate [10], [11] and endoscopy is not suitable for children. On the other hand, UBT has sensitivity and specificity approaching 100%, making it an appropriate noninvasive reference standard in children; thus, we included studies that relied on culture, histology and/or UBT to confirm *H. pylori* infection; and e) composition of the antigens included in commercial tests. In most cases antigen identity was unknown as this was considered proprietary information. Even after adjustments in a regression analysis no single component accounted for the wide variability observed (data not shown). Thus, ELISA tests in children showed high specificity and LR+ values, but low sensitivity and high LR− values. These findings have significant clinical implications, since a negative test would not be reliable for ensuring the absence of *H. pylori* infection. In other words, patients with negative ELISA results would present a fairly high chance of actually having past or current infection. Low sensitivity may be explained in part because of the weak or immature immune response observed in young children.

### Epidemiological and clinical implications

The interpretation of ELISA and WB tests in children depends on the purpose of the examination. If antibody detection is to be used for serologic-epidemiologic surveys, results suggest that both tests are useful tools. When antibody detection is used for pre-endoscopic screening, results suggest that a two-step serological approach should be followed; first, a serum ELISA-IgG to identify the majority of truly and potentially *H. pylori*-infected children and second, a WB-test to reduce false-positive ELISA results and eventually identify the specific antigens being recognized by the antibodies. If the purpose of antibody detection is to decide whether or not to treat *H. pylori* infection in children with severe dyspepsia or chronic abdominal pain, results suggest that antibody detection should not be used as the only justification for treatment. The serological test should be confirmed by another diagnostic test, in particular, histological examination or culture. In addition, the European Task Force [101] and NASPGN [10] recommend that screening of symptomatic children be performed by endoscopy to obtain a more complete differential diagnosis, e.g., pain, esophagitis, peptic ulceration, gastritis and *H. pylori* infection. Current serologic tests are not useful for monitoring eradication of infection after therapy since they cannot distinguish between current or past *H. pylori* infection, and the antibody titers usually remain positive several months after the infection has been eradicated. Recently, a new version of the commercial WB test, HelicoBlot® 2.1 (Genelabs Diagnostics, Singapore) has been marketed which includes an antigenic protein known as CIM (current infection marker). According to the manufacturer, the detection of anti-CIM IgG is highly predictive of active (current) *H. pylori* infection [83], [102]. Further studies are needed to confirm these findings.

In summary, high accuracy is provided by in-house ELISA tests for *H. pylori* infection in children. Advantages of ELISA are simplicity, minimal patient discomfort and the rapidity by which results can be obtained. On the other hand, also the WB test achieved high accuracy. However, WB is not widely available, perhaps due to its high cost, test handling requirements and differences in result interpretation. The main advantage of WB tests is the possibility of providing specific antigen profiles. Since the initial antibody response in children is principally to small molecular size antigens and, in chronic patients, to larger molecular size antigens, WB tests also provide information about the type of infecting strain as they can distinguish between CagA or VacA strains.

### Analysis of the heterogeneity

We investigated heterogeneity by stratifying the ELISA-IgG subgroup into commercial and in-house tests. The shape of the SROC curve (Figure 3) suggests that variability in the different thresholds used among studies could partially explain the heterogeneity [43], [44], [49]. The in-house tests showed higher accuracy, the DOR estimate was about five times greater than the value for commercial tests (224.8 vs 46.9); furthermore, with whole cell antigen the in-house test was six time greater (292.8 vs 46.9). However, considerable heterogeneity persisted in the commercial test group even after this stratification. The variability in the ELISA protocols may in-part explain this result. For example, within the commercial tests, 19 different protocols were described, including FlexSure®HP Serum Test (SmithKline Diagnostic, Palo Alto, CA, USA) and Pyloriset Screen (PS, Orion Diagnostica, Espoo, Finland) which are rapid blood tests, and others such as Cobas Core EIA® (Roche, Mannheim, Germany) and Enzygnost IgG (Behring, Marburg, Germany) which are based on conventional ELISA protocols. Heterogeneity could be related to differences in antigen source, composition, or level of purity. Diversity in the specificity of the immune response among populations due to differences in the infecting strains is also another factor that may explain variability. Yet another factor is the diversity in the ages of the children involved; several epidemiological studies have shown a significantly higher IgG response to *H. pylori* in older children [9], [10], [14], [31], [62], [78], [81]. Different times for obtaining samples could also affect results. Mitchel et al [14] have shown that in acutely infected children, there is an initial antibody response to small molecular size antigens and a later response to bigger proteins such as CagA; this could partially explain the increase of the sensitivity of the in-house test when using whole-cell as antigen versus recombinant proteins such as CagA. This suggests that recently infected children have not yet mounted an immune response to some *H. pylori* antigens. Finally, the comparison of a test against an imperfect reference standard could result in underestimation of test accuracy.

It is clear that there is a need to perform more studies with commercial *H. pylori* ELISA tests controlling as much as possible for all variables mentioned above, with a well designed and properly controlled protocol. In our meta-analysis we excluded studies with clinical data, but without standard confirmation (called class three reference). In general, since the sensitivity and specificity of ELISA test are widely variable, clinicians may have to rely on data developed in their own institutions or country, to produce clinically useful estimates of test accuracy, local adjustments particularly in the source of the antigen, i.e. use of whole-cell extracted from *H. pylori* strains isolated from the community must be validated in the populations [7], [10], [11], [78], [81], [101].

### Strengths and weaknesses of the review

An important strength of our study was its comprehensive search strategy, though it is possible that we may have missed some eligible studies. Screening, study selection and quality assessment were done independently by two reviewers. For some studies, we reduce the problem of missing data by contacting directly the authors. We also explore heterogeneity and potential publication bias in accordance with published guidelines. We analyzed data within specific subgroups to lessen the effect of heterogeneity.

We recognized some limitations of our study; we were able to include only English and Spanish language articles due to the linguistic abilities of our team, and this could have introduced selection bias to our results. Second, we did not address the effect of factors such as laboratory infrastructure, expertise with the technology test, and patient spectrum. Although, we used standard guidelines for reporting diagnostic accuracy of tests (STARD) to improve the quality of our analysis [46], our findings should be interpreted in the context of the quality of reporting and variability of the included studies. Unfortunately we were not able to evaluate the ELISA performance among different ages, because this information was not available in the majority of the included studies.

In conclusion, the evidence provided in this meta-analysis suggests that, at the current time, both WB test and in-house ELISA with whole-cell antigen are the most reliable tests for the diagnosis of *H. pylori* infection in children. Since the sensitivity and specificity of commercial ELISA tests are widely variable, clinicians may have to rely on tests developed in their own institution or country to warrant clinically useful results In particular, local adjustments in antigen source using *H. pylori* strains isolated from the community and validated for the population may be required.
